# Impact of glucose variability on the assessment of the glycemia risk index (GRI) and classic glycemic metrics

**DOI:** 10.1007/s12020-023-03511-7

**Published:** 2023-09-11

**Authors:** Paloma Pérez-López, Pablo Férnandez-Velasco, Pilar Bahillo-Curieses, Daniel de Luis, Gonzalo Díaz-Soto

**Affiliations:** 1 Endocrinology and Nutrition Department, Hospital Clínico Universitario Valladolid, Avenida Ramón y Cajal 3; CP, 47005 Valladolid, Spain; 2 Centro de Investigación en Endocrinología y Nutrición Clínica (CIENC), Universidad de Valladolid, Avenida Ramón y Cajal 3; CP, 47005 Valladolid, Spain; 3 Pediatrics Department, Hospital Clínico Universitario Valladolid, Avenida Ramón y Cajal 3; CP, 47005 Valladolid, Spain

**Keywords:** GRI, Glucose variability, GMI, Coefficient of variation, TIR

## Abstract

**Objective:**

To evaluate the impact of glucose variability on the relationship between the GRI and other glycemic metrics in a cohort of pediatric and adult patients with type 1 diabetes (T1D) using intermittent scanning continuous glucose monitoring (isCGM).

**Methods:**

We performed a cross-sectional study of 202 patients with T1D under intensive insulin treatment (25.2% CSII) using isCGM. Clinical, metabolic, and glycemic metrics were collected, and the GRI was calculated with its hypoglycemia (CHypo) and hyperglycemia (CHyper) components. The correlation between the GRI and other classical glycometrics in relation to the coefficient of variation (CV) was evaluated.

**Results:**

A total of 202 patients were included (53% male; 67.8% adults) with a mean age of 28.6 ± 15.7 years and 12.5 ± 10.9 years of T1D evolution (TIR 59.0 ± 17.0%; CV 39.8 ± 8.0%; GMI 7.3 ± 1.1%). The mean GRI was 54.0 ± 23.3 with a CHypo and CHyper component of 5.7 ± 4.8 and 23.4 ± 14.3, respectively. A strong negative correlation was observed between the GRI and TIR (*R* = −0.917; *R*^2^ = 0.840; *p* < 0.001), showing differences when dividing patients with low glycemic variability (CV < 36%) (*R* = −0.974; *R*^2^ = 0.948; *p* < 0.001) compared to those with greater CV instability (≥36%) (*R* = −0.885; *R*^2^ = 0.784; *p* < 0.001). The relationship of GRI with its two components was strongly positive with CHyper (*R* = 0.801; *R*^2^ = 0.641; *p* < 0.001) and moderately positive with CHypo (*R* = 0.398; *R*^2^ = 0.158; *p* < 0.001). When the GRI was evaluated with the rest of the classic glycemic metrics, a strong positive correlation was observed with HbA1c (*R* = 0.617; *R*^2^ = 0.380; *p* < 0.001), mean glucose (*R* = 0.677; *R*^2^ = 0.458; *p* < 0.001), glucose standard deviation (*R* = 0.778; *R*^2^ = 0.605; *p* < 0.001), TAR > 250 (*R* = 0.801; *R*^2^ = 0.641; *p* < 0.001), and TBR < 54 (*R* = 0.481; *R*^2^ = 0.231; *p* < 0.001).

**Conclusions:**

The GRI correlated significantly with all the glycemic metrics analyzed, especially with the TIR. Glycemic variability (GV) significantly affected the correlation of the GRI with other parameters and should be taken into consideration.

## Introduction

In recent years, the widespread use of continuous glucose monitoring (CGM) has led to a paradigm shift in glycemic control in patients with type 1 diabetes mellitus (T1D). An increasing number of studies have related the use of CGM to the improvement of metabolic control and quality of life and the reduction of long-term complications in patients with T1D [1–3]; however, the use of glycosylated hemoglobin A1c (HbA1c) continues to coexist with CGM parameters in clinical practice [4].

The limitations of HbA1c are well known: the lack of precision in laboratory measurement in common clinical situations (hemoglobinopathies, anemia, uremia, or pregnancy, among others), a half-life of around 3 months that does not allow short-term changes to be evaluated, the weak relationship with mean glucose at the individual level, as well as the low sensitivity to hypoglycemic events [4]. The CGM offers an interesting alternative, allowing a comprehensive assessment of interstitial blood glucose levels on a continuous basis, with the time in range (TIR) of 70–180 mg/dl, the measure with the most support at present [5]. In fact, its use has recently been recommended clinically and in trials, given the need for an evaluation beyond HbA1c of the different therapeutic measures in diabetes [6].

Due to both HbA1c and TIR being parameters of centrality, several studies have shown a strong negative relationship between the two variables [7, 8]. However, this correlation is directly influenced by the glycemic variability (GV) measured as the coefficient of variation (CV) in both T1D and type 2 diabetes mellitus (T2D) [9, 10]. In addition, TIR does not show sufficient sensitivity in the hypoglycemia range or in extreme glycemia values [6]. Therefore, simultaneous assessment of the different glycemic metrics of CGM is necessary for an adequate interpretation of metabolic control, with the consequent time-consuming workload for the professionals involved [6].

The recent development of the glycemia risk index (GRI) aims to solve some of these drawbacks by summarizing the overall quality of a given patient’s glycemic control in a single parameter. It arises from the analysis of the different scores given by 330 international experts in T1D to the CGM data of 225 insulin-treated patients. The GRI is calculated from the time below range (TBR) of <70 mg/dl and the time above range (TAR) of >180 mg/dl and gives greater weight to the extreme values of interstitial glucose [11]. This composite metric describes the quality of glycemia in CGM in a simple way and from a global point of view. Its simultaneous use with the “classic” glycometrics features may show some advantages; it encompasses metabolic control in a single parameter on a scale from 0 (very good control) to 100 (very poor control), allowing to prioritize or monitor the evolution of the same or different patients; the simple and easily automated calculation integrates into the Ambulatory Glucose Profile, an intuitive graphical representation and assessment with a clinical, not exclusively mathematical, background. In fact, a recent international consensus on CGM metrics for clinical trials suggested using the GRI for objective measures of glycaemic control derived from CGM [6].

Despite the possible advantages of integrating this new glucometry into routine clinical practice, its relationship with other glucose parameters, specifically the influence of GV on the GRI, has not yet been elucidated.

The present study aimed to evaluate the effect of glucose variability on the relationship between the GRI and other glycemic metrics and its clinical implications in a cohort of pediatric and adult patients with T1D using intermittently scanned continuous glucose monitoring (isCGM) treated with continuous subcutaneous insulin infusion (CSII) or multiple daily insulin injections (MDI).

## Methods

### Study population

We performed a cross-sectional study of a cohort of 202 patients with T1D on intensive insulin treatment and isCGM (FreeStyle Libre, Abbott Diabetes Care, Witney, UK) under follow-up in the Pediatric Endocrinology (<19 years old) and Endocrinology Departments at Hospital Clínico Universitario de Valladolid, Spain.

### Procedures

**Fig. 1:**
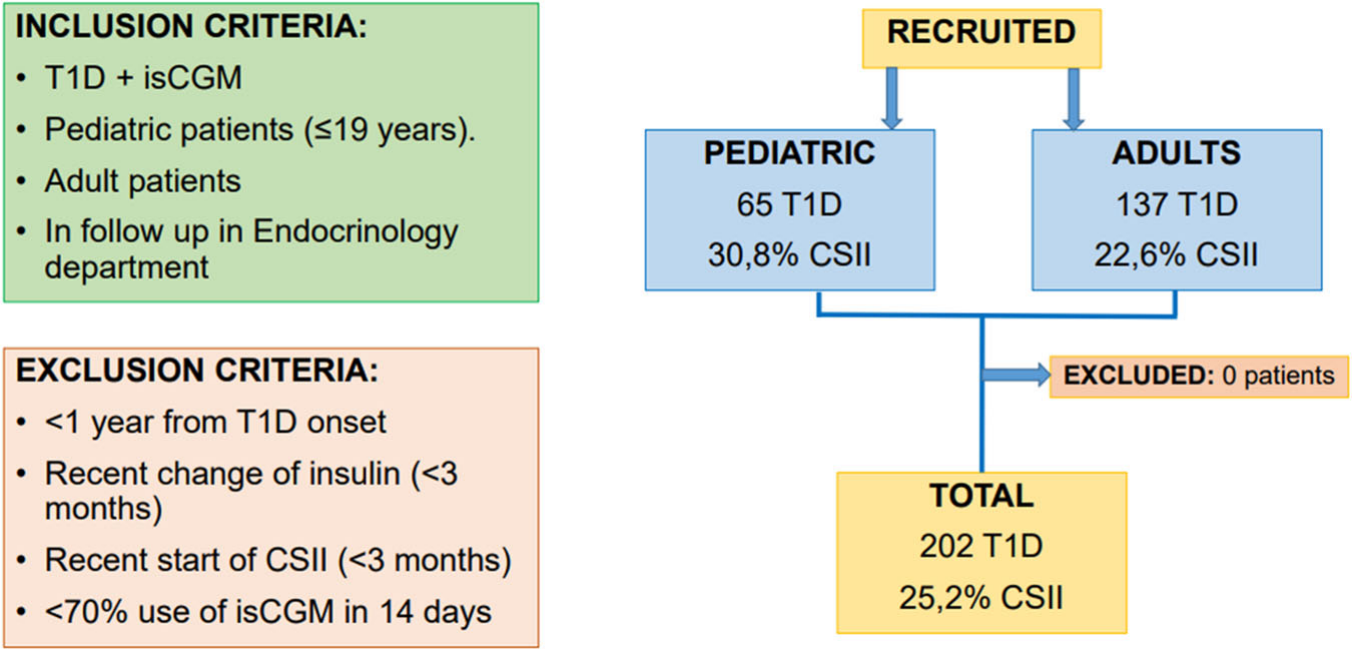
Flow chart showing inclusion and exclusion criteria and recruitment of patients for the study. T1D: Type 1 diabetes; isCGM: intermittently scanned continuous glucose monitoring; CSII: Continuous Subcutaneous Insulin Infusion

All patients with T1D and isCGM with a scheduled appointment between February 2019 and March 2019 were consecutively enrolled. Data on the use of the system and metabolic control was collected by analyzing downloaded device information. The last 14 days of isCGM prior to the patient’s visit were downloaded and analyzed, in all cases, after a minimum of 3 months of using the device. HbA1c was also measured between 7 and 10 days before the patient’s visit by turbidimetric inhibition immunoassay standardized to the National Glycohemoglobin Standardization Program (Roche Diagnostics, Geneva, Switzerland). Exclusion criteria were patients with inadequate use of the system (percentage of use less than 70% in the last 14 consecutive days) [6], or changes in their insulin regimen within the last 6 months (insulin type or CSII initiation) or who were less than 1 year after the onset of T1D. None of the patients met the exclusion criteria (Fig. 1)

Glucometric data of isCGM were defined as mean glucose (mg/dl), glucose management indicator (GMI) %, TIR (% of the time with glucose levels between 70–180 mg/dl), TAR (% of time above 180 mg/dl), TBR (% of the time below 70 mg/dl) and the number of daily scans. The GV was determined through CV% and standard deviation (SD) in mg/dl. TAR and TBR were also classified as very low glycemia level <54 mg/dL (<3.0 mmol/L)–TBR < 54; low glycemia level 54–70 mg/dL (3.0–3.9 mmol/L)–TBR 54–70; high glycemia level 181–250 mg/dL (10.1–13.9 mmol/L)–TAR 180–250; very high glycemia level > 250 mg/dL (>13.9 mmol/L)–TAR > 250. Hypoglycemia (CHypo) [TBR < 54 + (0.8 × TBR 54–70)] and hyperglycemia component (CHyper) [TAR > 250 + (0.5 × TAR 180–250)], as well as GRI [(3.0 × CHypo) + (1.6 × CHyper)] were calculated from original isCGM data, as previously described [11].

### Statistical analysis

The quantitative data were expressed as mean and SD if normally distributed, or median and interquartile range (25–75 percentile P25–75) if the TBR < 54 and TAR > 250. Qualitative variables were expressed in terms of percentages. The normal distribution of the variables was analyzed using the one-sample Kolmogorov–Smirnov test. The quantitative variables with normal distribution were analyzed using a bilateral Student’s *t*-test, and non-parametric variables were evaluated by using the Mann–Whitney U test. If necessary, categorical variables were assessed using the Chi-square test or the Fisher exact test. The association of quantitative variables was calculated using Pearson’s linear correlation coefficient. To evaluate high GV, a CV over 36% was selected as the cut-off point [6]. The *p*-values < 0.05 were considered statistically significant. The statistical package SPSS version 23.0 (SPSS Inc., Chicago, IL, UEA) and RStudio (2022) (RStudio: Integrated Development for R. RStudio, PBC, Boston, MA) were used for the analysis.

All patients signed an informed consent for their inclusion before participating in the study. The protocol was approved by the Clinical Research Ethics Committee of our Institution (PI 19-1390), and the study was conducted in accordance with the Declaration of Helsinki.

## Results

A total of 202 patients with T1D (53% male, 32.2% children, and adolescents defined as <19 years of age, 25.2% on CSII) were evaluated (Fig. 1). The mean age of the cohort was 28.6 ± 15.7 years, with 12.5 ± 10.9 years of diabetes evolution and a mean HbA1c of 7.3 ± 1.1%. The average number of daily scans was 10.4 ± 5.7, and the mean percentage of device use was 90.9 ± 10.3%. The glycemic metrics obtained were mean glucose (163.2 ± 33.3 mg/dl), mean SD (64.1 ± 19.0 mg/dl), GMI (7.3 ± 1.1%), CV (39.8 ± 8.0%), TIR (59.0 ± 17.0%), TBR < 54 1.0 [*P*25–75 0.0–3.0]%, TBR 54–70 (4.6 ± 3.4%), TAR 180–250 (22.0 ± 8.8%), and TAR > 250 9.0 [*P*25–75 3.75–18.0]%. The mean GRI was 54.0 ± 23.3, with CHypo and CHyper of 5.7 ± 4.8 and 23.4 ± 14.3, respectively (Table 1). Differences between adult and pediatric populations and CSII and MDI treatment are shown in Table 1. Pediatric patients and those with CSII treatment show better GRI, despite a higher CHypo than adults and MDI patients, respectively.

**Table 1:** Baseline features of total patients and adult versus pediatric and mutiple dosis of insuline versus CSII

	Total Patients	Adult Patients	Pediatric Patients	*p* value	CSII	MDI	*p* value
Number of patients	202	137	65	-	51	151	-
CSII (%)	25.2	22.6	30.8	ns	-	-	-
Gender (% women)	47.0	48.2	44.6	ns	51.0	45.7	ns
Mean age (years)	28.6 (15.7)	36.7 (12.6)	11.7 (3.3)	<0.01	26.3 (13.0)	29.4 (16.5)	NS
Years of evolution	12.5 (10.9)	17.8 (11.3)	4.9 (3.5)	<0.01	13.5 (10.7)	12.1 (11.0)	NS
Nº daily scans	10.4 (5.7)	9.5 (5.1)	12.5 (6.8)	<0.01	11.7 (7.6)	10.0 (5.0)	NS
% Sensor use	90.9 (10.3)	91.8 (9.2)	88.8 (12.5)	NS	90.8 (10.1)	90.9 (10.4)	NS
Mean HbA1C (%)	7.3 (1.1)	7.4 (1.1)	6.7 (0.6)	<0.01	7.0 (0.7)	7.3 (1.1)	<0.01
Mean glucose (mg/dl)	163.2 (33.3)	170.7 (35.3)	147.5 (21.9)	<0.01	154.2 (23.7)	166.2 (35.6)	<0.01
GMI (%)	7.3 (1.1)	7.5 (1.2)	6.8 (0.6)	<0.01	7.0 (0.7)	7.4 (1.2)	<0.01
% TIR (70–180 mg/dL)	59.0 (17.0)	55.4 (17.5)	66.5 (13.1)	<0.01	62.5 (11.5)	57.8 (18.3)	=0.033
% TBR (54–69 mg/dL)	4.6 (3.4)	4.0 (3.0)	5.9 (3.8)	<0.01	5.8 (3.2)	4.2 (3.4)	<0.01
* % TBR (<54 mg/dL)	1.0 [0.0–3.0]	1.0 [0.0–2.0]	1.0 [0.0–4.0]	NS	1.0 [0.0–3.0]	1.0 [0.0–3.0]	NS
% TAR (181–250 mg/dL)	22.0 (8.8)	24.3 (9.0)	17.2 (6.0)	<0.01	20.5 (6.5)	22.5 (9.4)	NS
* % TAR (>250 mg/dL)	9.0 [3.75–18.0]	13.0 [4.0–19.0]	6.0 [2.0–12.5]	<0.01	7.0 [4.0–13.0]	10.0 [3.0–19.0]	=0.012
SD (mg/dl)	64.1 (19.0)	64.6 (19.5)	62.9 (18.2)	ns	63.5 (13.5)	64.3 (20.6)	NS
CV (%)	39.8 (8.0)	38.6 (7.2)	42.4 (8.9)	<0.01	41.0 (6.6)	39.4 (8.4)	NS
GRI	54.0 (23.3)	56.8 (23.4)	48.0 (22.2)	=0.011	51.0 (15.3)	55.0 (25.4)	ns
Chypo	5.7 (4.8)	5.0 (4.5)	7.1 (5.1)	<0.01	6.5 (4.1)	5.4 (5.0)	<0.01
Chyper	23.4 (14.3)	26.5 (15.1)	16.8 (9.8)	<0.01	19.6 (10.6)	24.6 (15.2)	=0.042

Mean (Standard deviation) or * Median [Percentil25 − Percentil75]

*CSII* Continuous Subcutaneous Insulin Infusion, *TIR* time in range, *TAR* time above range, *TBR* time below range, *CV* coefficient of glycemic variability, *GMI* glucose management indicator, *SD* standard deviation, *GRI* glycemia risk index, *Chypo* hypoglycemia component, *Chyper* hyperglycemia component, *NS* not significant, *MDI* multiple daily insulin injections

When correlating GRI values and their CHyper and CHypo components with respect to classical glycometric parameters, a statistically significant correlation between GRI and classical glycemic metrics was observed. A strong GRI and TIR negative correlation was analyzed (*R* = −0.917; *R*^2^ = 0.840; *p* < 0.001), and a strong positive between GRI and HbA1c (*R* = 0.617; *R*^2^ = 0.380; *p* < 0.001), mean glucose (*R* = 0.677; *R*^2^ = 0.458; *p* < 0.001), SD (*R* = 0.778; *R*^2^ = 0.605; *p* < 0.001), GMI (*R* = 0.650; *R*^2^ = 0.422; *p* < 0.001), TBR < 54 (*R* = 0.481; *R*^2^ = 0.231; *p* < 0.001), TAR > 250 (*R* = 0.801; *R*^2^ = 0.641; *p* < 0.001), and CV (*R* = 605; *R*^2^ = 0.366; *p* < 0.001) were observed, as shown in Table 2.

**Table 2:** Correlation between classic glycometric parameters and GRI

Parámetros		GRI	CHypo	CHyper	HbA1c	Mean glucose	SD	GMI	TBR < 54	TBR 54–70	TIR 70−180	TAR 180−250	TAR > 250	CV
GRI	*R* *p*	1 -	0.398 <0.001	0.801 <0.001	0.617 <0.001	0.677 <0.001	0.778 <0.001	0.650 <0.001	0.481 <0.001	0.217 0.002	−0.917 <0.001	0.460 <0.001	0.801 <0.001	0.605 <0.001
CHypo	*R* *p*	0.398 <0.001	1 -	−0.223 <0.001	−0.221 0.002	−0.329 <0.001	0.256 <0.001	−0.278 <0.001	0.878 <0.001	0.877 <0.001	−0.041 NS	−0.335 <0.001	−0.145 0.039	0.711 <0.001
CHyper	*R* *p*	0.801 <0.001	−0.223 <0.001	1 -	0.818 <0.001	0.951 <0.001	0.650 <0.001	0.878 <0.001	−0.055 NS	−0.336 <0.001	−0.944 <0.001	0.676 <0.001	0.961 <0.001	0.176 0.012
HbA1c	*R* *p*	0.617 <0.001	−0.221 0.002	0.818 <0.001	1 -	0.837 <0.001	0.519 <0.001	0.821 <0.001	−0.730 NS	−0.313 <0.001	−0.774 <0.001	0.576 <0.001	0.775 <0.001	0.065 NS
Mean glucose	*R* *p*	0.677 <0.001	−0.329 <0.001	0.951 <0.001	0.837 <0.001	1 -	0.601 <0.001	0.932 <0.001	−0.153 0.030	−0.424 <0.001	−0.863 <0.001	0.616 <0.001	0.925 <0.001	0.064 NS
SD	*R* *p*	0.778 <0.001	0.256 <0.001	0.650 <0.001	0.519 <0.001	0.601 <0.001	1 -	0.583 <0.001	0.325 <0.001	0.123 NS	−0.718 <0.001	0.338 <0.001	0.662 <0.001	0.702 <0.001
GMI	*R* *p*	0.650 <0.001	−0.278 <0.001	0.878 <0.001	0.821 <0.001	0.932 <0.001	0.583 <0.001	1 -	−0.123 NS	−0.372 <0.001	−0.811 <0.001	0.554 <0.001	0.858 <0.001	0.107 NS
TBR < 54	*R* *p*	0.481 <0.001	0.878 <0.001	−0.055 NS	−0.730 NS	−0.153 0.030	0.325 <0.001	−0.123 NS	1 -	0.540 <0.001	−0.171 0.015	−0.193 0.006	0.006 NS	0.639 <0.001
TBR 54–70	*R* *p*	0.217 0.002	0.877 <0.001	−0.336 <0.001	−0.313 <0.001	−0.424 <0.001	0.123 NS	−0.372 <0.001	0.540 <0.001	1 -	0.099 NS	−0.394 <0.001	−0.261 <0.001	0.609 <0.001
TIR 70–180	*R* *p*	−0.917 <0.001	−0.041 NS	−0.944 <0.001	−0.774 <0.001	−0.863 <0.001	−0.718 <0.001	−0.811 <0.001	−0.171 0.015	0.099 NS	1 -	−0.721 <0.001	−0.877 <0.001	−0.347 <0.001
TAR 180–250	*R* *p*	0.460 <0.001	−0.335 <0.001	0.676 <0.001	0.576 <0.001	0.616 <0.001	0.338 <0.001	0.554 <0.001	−0.193 0.006	−0.394 <0.001	−0.721 <0.001	1 -	0.448 <0.001	−0.088 NS
TAR > 250	*R* *p*	0.801 <0.001	−0.145 0.039	0.961 <0.001	0.775 <0.001	0.925 <0.001	0.662 <0.001	0.858 <0.001	0.006 NS	−0.261 <0.001	−0.877 <0.001	0.448 <0.001	1 -	0.247 <0.001
CV	*R* *p*	0.605 <0.001	0.711 <0.001	0.176 0.012	0.065 NS	0.064 NS	0.702 <0.001	0.107 NS	0.639 <0.001	0.609 <0.001	−0.347 <0.001	−0.088 NS	0.247 <0.001	1 -

*GRI* glycemia risk index, *CHypo* component of hypoglycemia, *CHyper* component of hyperglycemia, *HbA1c* glycosylated hemoglobin A1c, *SD* standard deviation, *GMI* glucose management indicator, *TBR* time below range, *TIR* time in range, *TAR* time above range, *CV* coefficient of variation, *NS* not significant, *R* Pearson’s correlation coefficient, *p* *p* value

**Fig. 2:**
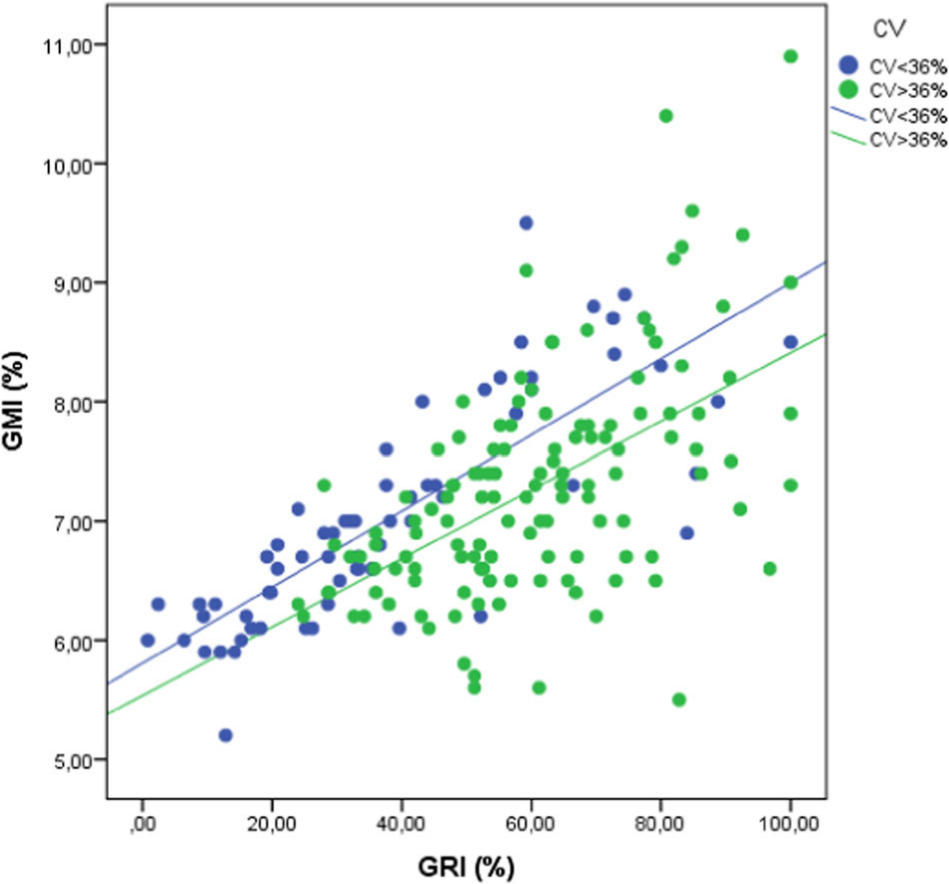
Scatter plot showing the correlation between gri and estimated HbA1c stratified according to the coefficient of variation. Those individuals with a coefficient of variation below 36% are shown in blue, while those with CV equal to or greater than 36% are shown in green. GMI: estimated glycosylated hemoglobin A1c or Glucose Management Indicator; GRI: Glycemia Risk Index; CV: coefficient of variation

When evaluating the relationship between the estimated HbA1c and GRI, a strong positive correlation was found (*R* = 0.650; *R*^2^ = 0.442; *p* < 0.001). This correlation was modified according to the degree of GV measured by the CV; a statistically significant better correlation was observed in those subjects with low glycemic variability (CV < 36%) (*R* = 0.812; *R*^2^ = 0.660; *p* < 0.001), compared to those with higher GV (CV ≥ 36%) (*R* = 0.553; *R*^2^ = 0.306; *p* < 0.001). Furthermore, it was observed that the slopes of both lines according to the CV run parallel to each other, with those individuals with greater glucose instability presenting lower HbA1c values for the same GRI value compared to those with CV < 36% (Fig. 2).

**Fig. 3:**
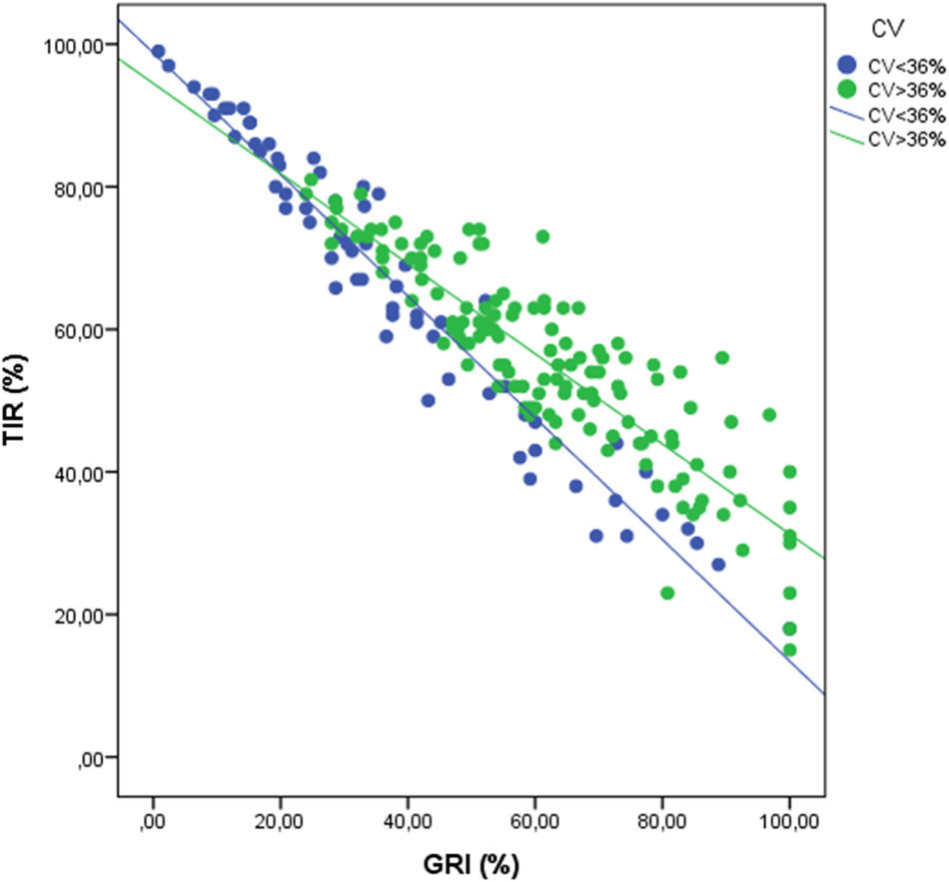
Scatter plot showing the correlation between GRI and TIR stratified according to the coefficient of variation. Those individuals with a coefficient of variation <36% are shown in blue, while those with CV ≥ 36% are shown in green. TIR Time in Range, GRI Glycemia Risk Index, CV Coefficient of Variation

Specifically, a strong negative correlation between the GRI and TIR was found (*R* = −0.917; *R*^2^ = 0.840; *p* < 0.001). This correlation showed differences according to the degree of GV measured by the CV, with a statistically significant better correlation being observed in those subjects with low GV (CV < 36%) (*R* = −0.974; *R*^2^ = 0.948; *p* < 0.001) versus those with higher GV (CV ≥ 36%) (*R* = −0.885; *R*^2^ = 0.784; *p* < 0.001) (Fig. 3). The cut-off point between both lines was obtained at a GRI value of 23 and a TIR of 78%. The correlation between the GRI and TIR remained stable when analyzing both the pediatric group (*R* = −0.929; *R*^2^ = 0.859; *p* < 0.001), adult group (*R* = −0.925; *R*^2^ = 0.855; *p* < 0.001), MDI (*R* = −0.924, *R*^2^ = 0.860; *p* < 0.001) or CSII (*R* = −0.865; *R*^2^ = 0.789; *p* < 0.001). However, the correlation between GRI and CHypo showed a better correlation in those subgroups with higher hypoglycemia risk, particularly the pediatric group (*R* = 0.724, *R*^2^ = 0.685, *p* < 0.001), than in adults (*R* = 0.322, *R*^2^ = 0.289, *p* < 0.001), respectively (Supplementary Infomation)

**Fig. 4:**
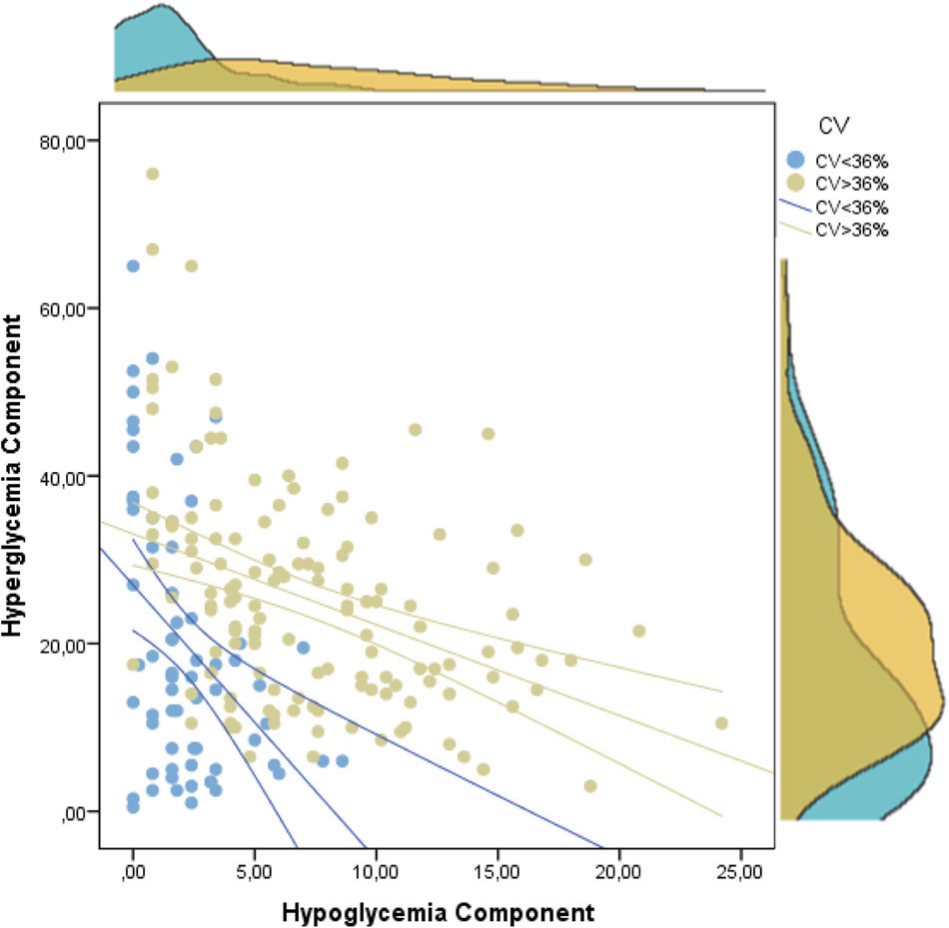
Scatter plot showing the correlation of the hypo and hyperglycemia components of the GRI. Those individuals with a coefficient of variation below 36% are shown in blue, while those with CV equal to or greater than 36% are shown in yellow. CV Coefficient of Variation

When analyzing the relationship between the CHypo and CHyper components of the GRI, a statistically significant negative, weak correlation was observed between both parameters (*R* = −0.223; *R*^2^ = 0.049; *p* < 0.001). Those individuals with CV ≥ 36% showed a heterogeneous distribution with higher CHypo values and greater CHyper variability, with respect to those with a CV below 36%, who presented a greater clustering around the hyperglycemia axis, with lower CHypo values (Fig. 4).

Finally, the results of the different glucometries were analyzed according to the CV, with significantly lower values of GRI (38.2 ± 23.8 vs. 61.7 ± 19.0; *p* < 0.001), CHypo (2.1 ± 2.0 vs. 7.4 ± 4.9; *p* < 0.001), CHyper (19.9 ± 16.1 vs. 25.1 ± 13.1; *p* = 0.024), GMI (7.0 ± 0.9 vs. 7.4 ± 1.1%; *p* = 0.039), SD (49.9 ± 13.0 vs. 71.0 ± 17.6 mg/dl; *p* < 0.001), TBR < 54 mg/dl (0.5 ± 0.8 vs. 2.7 ± 3.1%; *p* < 0.001), TBR 54–70 mg/dl (2.1 ± 1.7 vs. 5.9 ± 3.4%; *p* < 0.001), TAR > 250 mg/dl (8.6 ± 11.3 vs. 14.2 ± 11.6%; *p* = 0.001), and higher TIR values (66.1 ± 20.8 vs. 55.5 ± 13.5%; *p* < 0.001), with respect to those with greater glycemic instability (CV ≥ 36%), as shown in Table 3.

**Table 3:** Results of glycometric parameters stratified according to the coefficient of variation

PARAMETERS	CV	Mean (SD)	*p* value
GRI	<36 ≥36	38.2 (23.8) 61.7 (19.0)	<0.001
CHypo	<36 ≥36	2.1 (2.0) 7.4 (4.9)	<0.001
CHyper	<36 ≥36	19.9 (16.1) 25.1 (13.1)	0.024
HbA1c (%)	<36 ≥36	7.1 (1.0) 7.3 (1.0)	NS
Mean Glucose (mg/dL)	<36 ≥36	158.9 (32.7) 165.2 (33.5)	NS
SD (mg/dl)	<36 ≥36	49.9 (13.0) 71.0 (17.6)	<0.001
GMI (%)	<36 ≥36	7.0 (0.9) 7.4 (1.1)	0.039
* TBR < 54 mg/dL (%)	<36 ≥36	0.0 [0.0–1.0] 1.0 [0.0–4.0]	<0.001
TBR 54–70 mg/dL (%)	<36 ≥36	2.1 (1.7) 5.9 (3.4)	<0.001
TIR 70–180 mg/dL (%)	<36 ≥36	66.1 (20.8) 55.5 (13.5)	<0.001
TAR 180–250 mg/dL (%)	<36 ≥36	22.5 (12.6) 21.7 (6.3)	NS
* TAR > 250 mg/dL (%)	<36 ≥36	4.0 [1.0–15.0] 12.0 [6.0–18.0]	<0.001

Mean (Standard Deviation) or * Median [Percentil25 − Percentil75]

*GRI* glycemia risk index, *CHypo* component of hypoglycemia, *CHyper* component of hyperglycemia, *HbA1c* glycosylated hemoglobin A1c, *SD* standard deviation, *GMI* glucose management indicator, *TBR* time below range, *TIR* time in range, *TAR* time above range, *CV* coefficient of variation, *NS* not significant

## Discussion

The multiple glycometric data provided by CGM systems have allowed the development of new indices to measure the glycemic control of patients with T1D. Although HbA1c is the parameter with the greatest evidence for predicting chronic complications to date, it is insufficient to optimally assess the degree of glycemic control of a given individual, and there are already studies that relate the TIR to the risk of long-term complications [12, 13]. Nevertheless, the TIR alone shows certain limitations; it does not take into account whether the time out of range constitutes time in hypo- or hyperglycemia, it is not sensitive to time in hypoglycemia, and it does not give greater weight to the most extreme deviations from the TIR [2–4]. The large amount of data that the CGM provides demands a significant amount of time and effort from the professionals involved in the management of these patients [6, 14], requiring new metrics that synthesize all this available information.

The appearance of the GRI attempts to solve some of these drawbacks since it is a single parameter whose value ranges from 0 (best degree of glycemic control) to 100 (worst control), is actionable, calculated from a simple formula that gives greater weight to extreme glycemic values, is easily interpretable, and whose changes can be evaluated over time. Moreover, it arises with the support of many international experts in T1D [11]. The GRI uses only four parameters from the AGP report (TBR < 54, TBR 54–70, TAR 180–250, and TAR > 250 mg/dL) and can be easily applied in a wide variety of study designs and settings that use CGM to assess outcomes. Recently, a 14-day period of CGM data has been established as the most appropriate for the calculation of the GRI [15].

To date, published results extensively analyzing the correlation of GRI with other glycometric parameters correspond to data collected from clinical trials in adults with Dexcom G4 and G6 CGM systems (Dexcom Inc, San Diego, CA, USA). Therefore, to our knowledge, this is the first study that investigates the consistency of these relationships in an actual clinical practice setting using isCGM and a pediatric population. It also stratifies the GRI correlation results according to GV, whose influence has already been demonstrated in the relationship between TIR and HbA1c by different authors [9, 10].

Our results demonstrate the correlation between the different glycemic metrics most commonly used in clinical practice and the GRI and its components (Table 2). The statistically significant relationship between practically all the variables analyzed highlights the important interrelationship between the different parameters and emphasizes once again the difficulty of interpreting them independently. Logically, glycemic parameters could be grouped into measurements related to hyperglycemia (HbA1c, mean glucose, SD, GMI, TIR, TAR, and CHyper) and hypoglycemia (CV, TBR, and CHypo). The relationship between HbA1c and TIR was congruent with that found by Vigersky et al. (*R* = −0.84; *R*^2^ = 0.71; *p* < 0.001) [7] and Diaz-Soto et al. (*R* = −0.746; *R*^2^ = 0.557; *p* < 0.001) [10]. The GRI correlated significantly (and in most cases, strongly) with all the parameters analyzed, related to both hyper- and hypoglycemia, unlike its predecessors (glycosylated hemoglobin A1c and TIR), which did not correlate significantly or correlated weakly with hypoglycemia parameters. This ability of the GRI to better reflect changes in the area of hypoglycemia, which is derived from its formula (focused on the most extreme values of glycemia rather than on values of centrality), is one of the fundamental differences with respect to HbA1c and TIR and has been described in recent publications [16, 17]. Our correlations are similar to those described by Klonoff et al. [11] in the original GRI article, in which a smaller number of variables were analyzed, agreeing on the low correlation of TIR with parameters related to hypoglycemia such as TBR < 45 mg/dl (*R* = −0.11), TBR 70–45 mg/dl (*R* = 0.1), and CV (*R* = −0.27). Recently, a new study has evaluated the GRI correlation against CHypo and CHyper in T1D patients on an automated insulin delivery system. The results found a significant GRI correlation with TAR but not TBR [18]. These results are not surprising due to the low risk of hypoglycemia in the population evaluated even before the use of the automatic insulin system (TBR around 3.9%). In fact, our findings are in line with these results. Those subgroups of patients at higher risk of hypoglycemia, especially the pediatric group, showed significantly stronger correlations of GRI with CHypo/TBR. Moreover, following this approach, we should consider the lack of usefulness of the GRI in those subgroups of patients with practically nonexistent TBR or TAR.

The stratification of the correlation between the GRI and GMI according to the CV showed two lines with parallel slopes, with those patients with a CV ≥ 36% belonging to the line that runs along the lower part of Fig. 2. According to this, for the same HbA1c value, those patients with greater CV instability showed higher GRI values than those with lower GV. For example, for an estimated HbA1c of 7%, those individuals with a CV < 36% presented a mean GRI of 39.7, while those with a CV ≥ 36% showed a GRI of 48.7 (a GRI 18.5% higher). This parallel relationship between the two lines may be explained by the sensitivity of the CV in assessing an individual’s risk of hypoglycemia [18], the most penalized component in calculating the GRI. This parallel relationship is also different from the previously published relationships of the CV in relation to the TIR and GMI, where the lines crossed each other [9, 10], demonstrating the weighting provided by the effect of the GV all along the GRI calculation.

The relationship between the GRI and TIR in our work is practically analogous to the results published by Klonoff et al. in the original GRI article (*R* = −0.910; *p* < 0.001) [11]. Moreover, this correlation was maintained for patients with MDI and CSII. In the present work, we also found a similar correlation between these two parameters in the pediatric and adult groups, which had not been previously studied. When stratifying the data according to the CV, it was observed that both regression lines intersected at a value of GRI = 23 and TIR = 78%. For a TIR value greater than 78%, the higher the CV, the lower the GRI, while when the TIR is lower than 78%, the GRI is higher as the CV increases (Fig. 3). This relationship can be partially explained by the distribution of the CHypo and CHyper components that make up the GRI (Fig. 4) and explains much of the variability in the correlation between the GRI and TIR. When analyzing the relationship between the CHypo and CHyper components of the GRI according to GV, it was observed that those with lower GV presented a greater clustering around the hyperglycemia axis. In contrast, those with a high CV showed a greater component of hypoglycemia and variable of hyperglycemia, supporting the relationship between CV and CHypo [19]. A recent study on an automated insulin delivery system in adults supports our findings because its low global CV showed a GRI clustering around the hyperglycemia axis [18]

Finally, when analyzing the different glycometric parameters according to a CV greater or less than 36% (Table 3), significant differences were observed for all glycometric parameters except HbA1c, mean glucose and TAR 180–250. As for the TIR, those patients with greater variability showed a decrease of 11% with respect to those with CV < 36%; however, for the GRI, the difference was 27 percentiles more in the group with higher variability, which highlights the greater weight of variability (and, ultimately, of hypoglycemia) in this new index. The fact that significant differences were found for the GMI and not for HbA1c (given that the mean and SD values are similar) seems to be due to the study’s sample size. As shown in Table 3, those patients with a CV ≥ 36% presented greater GRI and lower TIR, with a marked component of hypoglycemia and a tendency to greater hyperglycemia than those with low GV.

Limitations of the present study include the relatively small sample size compared to large data studies; however, this is a real-life cohort with stable control and comprehensive knowledge of glycometric and clinical variables with a single CGM system. The non-incorporation of TIR in the GRI calculation can be seen as a potential limitation, being the only CGM parameter related to long-term complications at present [11]; however, the high correlation between TIR and GRI suggests that a similar correlation exists between GRI and the existence of long-term complications as recent studies support [20, 21]. More studies are needed to relate this parameter to future complications, its effect on the quality of life of patients with T1D, and its evaluation in other subpopulations (T2D, hospitalized patients). Finally, some studies have shown higher TBR in FreeStyle Libre isCGM users [22]. This could increase CHypo in our investigation. However, the use of the same isCGM model and version throughout the study in all patients ensures the representativeness of our results. As strengths, it is worth highlighting the results, in line with those previously published on the relationship of the GRI with other glycometrics [11], as well as the influence of the CV on the relationship between the different parameters of glycemic control in adult and pediatric patients in a non-selected population on different treatments [9, 10].

In conclusion, the GRI correlated significantly with all the glycometric parameters analyzed, related to both hypo- and hyperglycemia and especially closely with TIR. GV measured as the CV significantly affected the correlation of GRI with TIR and Glycosylated Hemoglobin A1c and should be considered when metabolic control is assessed.

### Supplementary information

Supplementary Tables
