# Supplementary material for: Impact of glucose variability on the assessment of the glycemia risk index (GRI) and classic glycemic metrics
Source: Endocrine. 2023 Sep 11;82(3):560–8. doi: 10.1007/s12020-023-03511-7 (PMC10618378; doi:10.1007/s12020-023-03511-7)
Supplement: Supplementary file 1 — Supplementary Tables [file 12020_2023_3511_MOESM1_ESM.docx]

Table 1: CORRELATION BETWEEN CLASSIC GLYCOMETRIC PARAMETERS AND GRI: ADULT GROUP

| PARAMETERS |  | GRI | CHypo | | CHyper | HbA1c | Mean glucose | SD | GMI | TBR <54 | TBR 54-70 | TIR  70-180 | TAR 180 -250 | TAR >250 | CV |
| --- | --- | --- | --- | --- | --- | --- | --- | --- | --- | --- | --- | --- | --- | --- | --- |
| GRI | **R**  p | 1  - | 0,322  <0,001 | 0,832  <0,001 | | 0,642  <0,001 | 0,712  <0,001 | 0,750  <0,001 | 0,657  <0,001 | 0,407  <0,001 | 0,145  NS | -0,925  <0,001 | 0,471  <0,001 | 0,818  <0,001 | 0,583  <0,001 |
| CHypo | **R**  p | 0,322  <0,001 | 1  - | -0,248  <0,003 | | -0,216  0,013 | -0,330  <0,001 | 0,213  0,013 | -0,278  <0,001 | 0,894  <0,001 | 0,865  <0,001 | 0,011  NS | -0,321  <0,001 | -0,180  0,035 | 0,708  <0,001 |
| CHyper | **R**  p | 0,832  <0,001 | -0,248  <0,003 | 1  - | | 0,800  <0,001 | 0,944  <0,001 | 0,631  <0,001 | 0,863  <0,001 | -0,098  NS | -0,353  <0,001 | -0,948  <0,001 | 0,628  <0,001 | 0,961  <0,001 | 0,181  0,035 |
| HbA1c | **R**  p | 0,642  <0,001 | -0,216  0,013 | 0,800  <0,001 | | 1  - | 0,823  <0,001 | 0,524  <0,001 | 0,800  <0,001 | -0,077  NS | -0,311  <0,001 | -0,765  <0,001 | 0,504  <0,001 | 0,767  <0,001 | 0,111  NS |
| Mean glucose | **R**  p | 0,712  <0,001 | -0,330  <0,001 | 0,944  <0,001 | | 0,823  <0,001 | 1  - | 0,600  <0,001 | 0,929  <0,001 | -0,178  0,038 | -0,416  <0,001 | -0,866  <0,001 | 0,535  <0,001 | 0,928  <0,001 | 0,088  NS |
| SD | **R**  p | 0,750  <0,001 | 0,213  0,013 | 0,631  <0,001 | | 0,524  <0,001 | 0,600  <0,001 | 1  - | 0,579  <0,001 | 0,262  0,002 | 0,105  NS | -0,692  <0,001 | 0,328  <0,001 | 0,631  <0,001 | 0,672  <0,001 |
| GMI | **R**  p | 0,657  <0,001 | -0,278  <0,001 | 0,863  <0,001 | | 0,800  <0,001 | 0,929  <0,001 | 0,579  <0,001 | 1  - | -0,147  NS | -0,354  <0,001 | -0,803  <0,001 | 0,479  <0,001 | 0,850  <0,001 | 0,121  NS |
| TBR <54 | **R**  p | 0,407  <0,001 | 0,894  <0,001 | -0,098  NS | | -0,077  NS | -0,178  0,038 | 0,262  0,002 | -0,147  NS | 1  - | 0,549  <0,001 | -0,111  NS | -0,197  0,006 | -0,046  NS | 0,633  <0,001 |
| TBR 54-70 | **R**  p | 0,145  NS | 0,865  <0,001 | -0,353  <0,001 | | -0,311  <0,001 | -0,416  <0,001 | 0,105  NS | -0,354  <0,001 | 0,549  <0,001 | 1  - | 0,145  NS | -0,378  <0,001 | -0,284  <0,001 | 0,612  <0,001 |
| TIR 70-180 | **R**  p | -0,925  <0,001 | 0,011  NS | -0,948  <0,001 | | -0,765  <0,001 | -0,866  <0,001 | -0,692  <0,001 | -0,803  <0,001 | -0,111  NS | 0,145  NS | 1  - | -0,703  <0,001 | -0,873  <0,001 | -0,334  <0,001 |
| TAR 180-250 | **R**  p | 0,471  <0,001 | -0,321  <0,001 | 0,628  <0,001 | | 0,504  <0,001 | 0,535  <0,001 | 0,328  <0,001 | 0,479  <0,001 | -0,197  0,006 | -0,378  <0,001 | -0,703  <0,001 | 1  - | 0,390  <0,001 | -0,073  NS |
| TAR >250 | **R**  p | 0,818  <0,001 | -0,180  0,035 | 0,961  <0,001 | | 0,767  <0,001 | 0,928  <0,001 | 0,631  <0,001 | 0,850  <0,001 | -0,046  NS | -0,284  <0,001 | -0,873  <0,001 | 0,390  <0,001 | 1  - | 0,240  <0,001 |
| CV | **R**  p | 0,583  <0,001 | 0,708  <0,001 | 0,181  0,035 | | 0,111  NS | 0,088  NS | 0,672  <0,001 | 0,121  NS | 0,633  <0,001 | 0,612  <0,001 | -0,334  <0,001 | -0,073  NS | 0,240  <0,001 | 1  - |

GRI: Glycemia Risk Index; CHypo: Component of Hypoglycemia; CHyper: Component of Hyperglycemia; HbA1c: Glycosylated hemoglobin A1c; SD: Standard deviation; GMI: Glucose Management Indicator; TBR: Time Below Range; TIR: Time in Range; TAR: Time Above Range; CV: Coefficient of Variation; NS: not significant; R: Pearson's correlation coefficient; p: p-value

Table 2: CORRELATION BETWEEN CLASSIC GLYCOMETRIC PARAMETERS AND GRI: PEDIATRIC GROUP

| PARAMETERS |  | GRI | CHypo | | CHyper | HbA1c | Mean glucose | SD | GMI | TBR <54 | TBR 54-70 | TIR  70-180 | TAR 180 -250 | TAR >250 | CV |
| --- | --- | --- | --- | --- | --- | --- | --- | --- | --- | --- | --- | --- | --- | --- | --- |
| GRI | **R**  p | 1  - | 0,742  <0,001 | 0,735  <0,001 | | 0,524  <0,001 | 0,546  <0,001 | 0,869  <0,001 | 0,591  <0,001 | 0,755  <0,001 | 0,536  <0,001 | -0,929  <0,001 | 0,319  0,010 | 0,771  <0,001 | 0,856  <0,001 |
| CHypo | **R**  p | 0,742  <0,001 | 1  - | 0,092  NS | | 0,013  NS | -0,135  NS | 0,438  <0,001 | -0,076  NS | 0,864  <0,001 | 0,871  <0,001 | -0,467  <0,001 | -0,193  NS | -0,184  NS | 0,704  <0,001 |
| CHyper | **R**  p | 0,735  <0,001 | 0,092  NS | 1  - | | 0,763  <0,001 | 0,948  <0,001 | 0,849  <0,001 | 0,903  <0,001 | 0,251  0,044 | -0,088  NS | -0,909  <0,001 | 0,668  <0,001 | 0,960  <0,001 | 0,560  <0,001 |
| HbA1c | **R**  p | 0,524  <0,001 | 0,013  NS | 0,763  <0,001 | | 1  - | 0,766  <0,001 | 0,661  <0,001 | 0,787  <0,001 | -0,108  NS | -0,083  NS | -0,682  <0,001 | 0,568  <0,001 | 0,710  <0,001 | 0,365  0,003 |
| Mean glucose | **R**  p | 0,546  <0,001 | -0,135  NS | 0,948  <0,001 | | 0,766  <0,001 | 1  - | 0,736  <0,001 | 0,913  <0,001 | 0,063  NS | -0,293  0,018 | -0,780  <0,001 | 0,715  <0,001 | 0,879  <0,001 | 0,356  0,004 |
| SD | **R**  p | 0,869  <0,001 | 0,438  <0,001 | 0,849  <0,001 | | 0,661  <0,001 | 0,736  <0,001 | 1  - | 0,730  <0,001 | 0,528  <0,001 | 0,236  NS | -0,902  <0,001 | 0,430  <0,001 | 0,866  <0,001 | 0,864  <0,001 |
| GMI | **R**  p | 0,591  <0,001 | -0,076  NS | 0,903  <0,001 | | 0,787  <0,001 | 0,913  <0,001 | 0,730  <0,001 | 1  - | 0,109  NS | -0,261  0,044 | -0,773  <0,001 | 0,651  <0,001 | 0,854  <0,001 | 0,417  0,001 |
| TBR <54 | **R**  p | 0,755  <0,001 | 0,864  <0,001 | 0,251  0,044 | | 0,108  NS | 0,063  NS | 0,528  <0,001 | 0,109  NS | 1  - | 0,505  <0,001 | -0,533  <0,001 | -0,076  NS | 0,333  0,007 | 0,663  <0,001 |
| TBR 54-70 | **R**  p | 0,536  <0,001 | 0,871  <0,001 | -0,088  NS | | -0,083  NS | -0,293  0,018 | 0,236  NS | -0,261  0,044 | 0,505  <0,001 | 1  - | -0,280  0,024 | 0,256  0,039 | -0,009  NS | 0,559  <0,001 |
| TIR 70-180 | **R**  p | -0,929  <0,001 | -0,467  <0,001 | -0,909  <0,001 | | -0,682  <0,001 | -0,780  <0,001 | -0,902  <0,001 | -0,773  <0,001 | -0,533  <0,001 | -0,280  0,041 | 1  - | -0,617  <0,001 | -0,868  <0,001 | -0,760  <0,001 |
| TAR 180-250 | **R**  p | 0,319  0,010 | -0,193  NS | 0,668  <0,001 | | 0,568  <0,001 | 0,715  <0,001 | 0,430  <0,001 | 0,651  <0,001 | -0,076  NS | -0,256  0,039 | -0,617  <0,001 | 1  - | 0,431  <0,001 | 0,180  NS |
| TAR >250 | **R**  p | 0,771  <0,001 | 0,184  NS | 0,960  <0,001 | | 0,710  <0,001 | 0,879  <0,001 | 0,866  <0,001 | 0,854  <0,001 | 0,333  0,007 | -0,009  NS | -0,868  <0,001 | 0,431  <0,001 | 1  - | 0,611  <0,001 |
| CV | **R**  p | 0,856  <0,001 | 0,704  <0,001 | 0,560  <0,001 | | 0,365  0,003 | 0,356  0,004 | 0,864  <0,001 | 0,417  0,001 | 0,663  <0,001 | 0,559  <0,001 | -0,760  <0,001 | 0,180  NS | 0,611  <0,001 | 1  - |

GRI: Glycemia Risk Index; CHypo: Component of Hypoglycemia; CHyper: Component of Hyperglycemia; HbA1c: Glycosylated hemoglobin A1c; SD: Standard deviation; GMI: Glucose Management Indicator; TBR: Time Below Range; TIR: Time in Range; TAR: Time Above Range; CV: Coefficient of Variation; NS: not significant; R: Pearson's correlation coefficient; p: p-value

Table 3: CORRELATION BETWEEN CLASSIC GLYCOMETRIC PARAMETERS AND GRI: MDI GROUP

| PARAMETERS |  | GRI | CHypo | | CHyper | HbA1c | Mean glucose | SD | GMI | TBR <54 | TBR 54-70 | TIR  70-180 | TAR 180 -250 | TAR >250 | CV |
| --- | --- | --- | --- | --- | --- | --- | --- | --- | --- | --- | --- | --- | --- | --- | --- |
| GRI | **R**  p | 1  - | 0,433  <0,001 | 0,815  <0,001 | | 0,629  <0,001 | 0,684  <0,001 | 0,793  <0,001 | 0,662  <0,001 | 0,486  <0,001 | 0,278  0,001 | -0,924  <0,001 | 0,475  <0,001 | 0,807  <0,001 | 0,655  <0,001 |
| CHypo | **R**  p | 0,433  <0,001 | 1  - | -0,161  0,048 | | -0,158  NS | -0,277  0,001 | 0,293  <0,001 | -0,215  0,009 | 0,896  <0,001 | 0,883  <0,001 | -0,098  NS | -0,279  0,001 | -0,090  NS | 0,743  <0,001 |
| CHyper | **R**  p | 0,815  <0,001 | -0,161  0,048 | 1  - | | 0,810  <0,001 | 0,947  <0,001 | 0,669  <0,001 | 0,876  <0,001 | -0,035  NS | -0,258  0,001 | -0,945  <0,001 | 0,665  <0,001 | 0,960  <0,001 | 0,238  0,003 |
| HbA1c | **R**  p | 0,629  <0,001 | -0,158  NS | 0,810  <0,001 | | 1  - | 0,833  <0,001 | 0,535  <0,001 | 0,820  <0,001 | -0,056  NS | -0,228  0,006 | -0,771  <0,001 | 0,561  <0,001 | 0,767  <0,001 | 0,115  NS |
| Mean glucose | **R**  p | 0,684  <0,001 | -0,277  0,001 | 0,947  <0,001 | | 0,833  <0,001 | 1  - | 0,611  <0,001 | 0,937  <0,001 | -0,142  NS | -0,358  <0,001 | -0,859  <0,001 | 0,594  <0,001 | 0,923  <0,001 | 0,109  NS |
| SD | **R**  p | 0,793  <0,001 | 0,293  <0,001 | 0,669  <0,001 | | 0,535  <0,001 | 0,611  <0,001 | 1  - | 0,591  <0,001 | 0,329  <0,001 | 0,189  0,021 | -0,737  <0,001 | 0,361  <0,001 | 0,673  <0,001 | 0,701  <0,001 |
| GMI | **R**  p | 0,662  <0,001 | -0,215  0,009 | 0,876  <0,001 | | 0,820  <0,001 | 0,937  <0,001 | 0,591  <0,001 | 1  - | -0,105  NS | -0,294  <0,001 | -0,807  <0,001 | 0,513  <0,001 | 0,865  <0,001 | 0,149  NS |
| TBR <54 | **R**  p | 0,486  <0,001 | 0,896  <0,001 | -0,035  NS | | -0,056  NS | -0,142  NS | 0,329  <0,001 | -0,105  NS | 1  - | 0,583  <0,001 | -0,193  0,018 | -0,156  NS | 0,016  NS | 0,659  <0,001 |
| TBR 54-70 | **R**  p | 0,278  0,001 | 0,883  <0,001 | -0,258  0,001 | | -0,228  0,006 | -0,358  <0,001 | 0,189  0,021 | -0,294  <0,001 | 0,583  <0,001 | 1  - | 0,024  NS | -0,347  <0,001 | -0,182  0,025 | 0,663  <0,001 |
| TIR 70-180 | **R**  p | -0,924  <0,001 | -0,098  NS | -0,945  <0,001 | | -0,771  <0,001 | -0,859  <0,001 | -0,737  <0,001 | -0,807  <0,001 | -0,193  0,018 | 0,024  NS | 1  - | -0,719  <0,001 | -0,872  <0,001 | -0,411  <0,001 |
| TAR 180-250 | **R**  p | 0,475  <0,001 | -0,279  0,001 | 0,665  <0,001 | | 0,561  <0,001 | 0,594  <0,001 | 0,361  <0,001 | 0,513  <0,001 | -0,156  NS | -0,347  <0,001 | -0,719  <0,001 | 1  - | 0,428  <0,001 | -0,033  NS |
| TAR >250 | **R**  p | 0,807  <0,001 | -0,090  NS | 0,960  <0,001 | | 0,767  <0,001 | 0,923  <0,001 | 0,673  <0,001 | 0,865  <0,001 | 0,016  NS | -0,182  0,025 | -0,872  <0,001 | 0,428  <0,001 | 1  - | 0,300  <0,001 |
| CV | **R**  p | 0,655  <0,001 | 0,743  <0,001 | 0,238  0,003 | | 0,115  NS | 0,109  NS | 0,701  <0,001 | 0,149  NS | 0,659  <0,001 | 0,663  <0,001 | -0,411  <0,001 | -0,033  NS | 0,300  <0,001 | 1  - |

MDI: Multiple Daily Insulin Injections; GRI: Glycemia Risk Index; CHypo: Component of Hypoglycemia; CHyper: Component of Hyperglycemia; HbA1c: Glycosylated hemoglobin A1c; SD: Standard deviation; GMI: Glucose Management Indicator; TBR: Time Below Range; TIR: Time in Range; TAR: Time Above Range; CV: Coefficient of Variation; NS: not significant; R: Pearson's correlation coefficient; p: p-value

Table 4: CORRELATION BETWEEN CLASSIC GLYCOMETRIC PARAMETERS AND GRI: CSII GROUP

| PARAMETERS |  | GRI | CHypo | | CHyper | HbA1c | Mean glucose | SD | GMI | TBR <54 | TBR 54-70 | TIR  70-180 | TAR 180 -250 | TAR >250 | CV |
| --- | --- | --- | --- | --- | --- | --- | --- | --- | --- | --- | --- | --- | --- | --- | --- |
| GRI | **R**  p | 1  - | 0,286  0,042 | 0,713  <0,001 | | 0,522  <0,001 | 0,611  <0,001 | 0,678  <0,001 | 0,525  <0,001 | 0,470  <0,001 | 0,037  NS | -0,865  <0,001 | 0,323  0,021 | 0,761  <0,001 | 0,382  0,006 |
| CHypo | **R**  p | 0,286  0,042 | 1  - | -0,467  0,001 | | -0,485  <0,001 | -0,547  <0,001 | 0,071  NS | -0,563  <0,001 | 0,825  <0,001 | 0,863  <0,001 | 0,199  NS | -0,590  <0,001 | -0,355  0,011 | 0,549  <0,001 |
| CHyper | **R**  p | 0,713  <0,001 | -0,467  0,001 | 1  - | | 0,837  <0,001 | 0,965  <0,001 | 0,566  <0,001 | 0,877  <0,001 | -0,171  NS | -0,595  <0,001 | -0,943  <0,001 | 0,722  <0,001 | 0,965  <0,001 | -0,057  NS |
| HbA1c | **R**  p | 0,522  <0,001 | -0,485  <0,001 | 0,837  <0,001 | | 1  - | 0,839  <0,001 | 0,450  0,001 | 0,796  <0,001 | -0,171  NS | -0,623  <0,001 | -0,770  <0,001 | 0,645  <0,001 | 0,793  <0,001 | -0,120  NS |
| Mean glucose | **R**  p | 0,611  <0,001 | -0,547  <0,001 | 0,965  <0,001 | | 0,839  <0,001 | 1  - | 0,569  <0,001 | 0,892  <0,001 | -0,241  NS | -0,660  <0,001 | -0,879  <0,001 | 0,720  <0,001 | 0,922  <0,001 | -0,093  NS |
| SD | **R**  p | 0,678  <0,001 | 0,071  NS | 0,566  <0,001 | | 0,450  0,001 | 0,569  <0,001 | 1  - | 0,522  <0,001 | 0,307  0,029 | -0,160  NS | -0,604  <0,001 | 0,190  NS | 0,629  <0,001 | 0,742  <0,001 |
| GMI | **R**  p | 0,525  <0,001 | -0,563  <0,001 | 0,877  <0,001 | | 0,796  <0,001 | 0,892  <0,001 | 0,552  <0,001 | 1  - | -0,271  NS | -0,652  <0,001 | -0,815  <0,001 | 0,811  <0,001 | 0,784  <0,001 | -0,068  NS |
| TBR <54 | **R**  p | 0,470  <0,001 | 0,825  <0,001 | -0,171  NS | | -0,171  NS | -0,241  NS | 0,307  0,029 | -0,271  NS | 1  - | 0,427  0,002 | -0,045  NS | -0,414  0,003 | -0,055  NS | 0,558  <0,001 |
| TBR 54-70 | **R**  p | 0,037  NS | 0,863  <0,001 | -0,595  <0,001 | | -0,623  <0,001 | -0,660  <0,001 | -0,160  NS | -0,652  <0,001 | 0,427  0,002 | 1  - | 0,359  0,010 | -0,575  <0,001 | -0,519  <0,001 | 0,380  0,006 |
| TIR 70-180 | **R**  p | -0,865  <0,001 | 0,199  NS | -0,943  <0,001 | | -0,770  <0,001 | -0,879 <0,001 | -0,604  <0,001 | -0,815  <0,001 | -0,045  NS | 0,359  0,010 | 1  - | -0,711  <0,001 | -0,898  <0,001 | -0,069  NS |
| TAR 180-250 | **R**  p | 0,323  0,021 | -0,590  <0,001 | 0,722  <0,001 | | 0,645  <0,001 | 0,720  <0,001 | 0,190  NS | 0,811  <0,001 | -0,414  0,003 | -0,575  <0,001 | -0,711  <0,001 | 1  - | 0,515  <0,001 | -0,347  0,013 |
| TAR >250 | **R**  p | 0,761  <0,001 | -0,355  0,011 | 0,965  <0,001 | | 0,793  <0,001 | 0,922  <0,001 | 0,629  <0,001 | 0,784  <0,001 | -0,055  NS | -0,519  <0,001 | -0,898  <0,001 | 0,515  <0,001 | 1  - | 0,061  NS |
| CV | **R**  p | 0,382  0,006 | 0,549  <0,001 | -0,057  NS | | -0,120  NS | -0,093  NS | 0,742  <0,001 | -0,068  NS | 0,558  <0,001 | 0,380  0,006 | -0,069  NS | -0,347  0,013 | 0,061  NS | 1  - |

CSII: Continuous Subcutaneous Insulin Infusion; GRI: Glycemia Risk Index; CHypo: Component of Hypoglycemia; CHyper: Component of Hyperglycemia; HbA1c: Glycosylated hemoglobin A1c; SD: Standard deviation; GMI: Glucose Management Indicator; TBR: Time Below Range; TIR: Time in Range; TAR: Time Above Range; CV: Coefficient of Variation; NS: not significant; R: Pearson's correlation coefficient; p: p-value

**RECRUITED**

**ADULTS**

137 T1D

22,6% CSII

**TOTAL**

202 T1D

25,2% CSII

**EXCLUDED: 0 patients**
